# Non-coding RNAs and epithelial mesenchymal transition in cancer: molecular mechanisms and clinical implications

**DOI:** 10.1186/s13046-022-02488-x

**Published:** 2022-09-16

**Authors:** Hashem Khanbabaei, Saeedeh Ebrahimi, Juan Luis García-Rodríguez, Zahra Ghasemi, Hossein Pourghadamyari, Milad Mohammadi, Lasse Sommer Kristensen

**Affiliations:** 1 Department of Radiologic Technology, Faculty of Allied Medicine, Kerman University of Medical Sciences, Mostafa Khomeyni St., Kerman, 7616911333 Iran; 2 Department of Medical Microbiology (Bacteriology & Virology), Afzalipour Faculty of Medicine, Kerman University of Medical Sciences, Kerman, Iran; 3 Department of Biomedicine, Aarhus University, Aarhus, Denmark; 4 Department of Molecular Genetics, Faculty of Modern Sciences, Tehran Medical Sciences Branch, Islamic Azad University, Tehran, Iran; 5 Department of Clinical Biochemistry, Afzalipour School of Medicine, Kerman University of Medical Sciences, Kerman, Iran; 6 Research Center for Hydatid Disease in Iran, Kerman University of Medical Sciences, Kerman, Iran; 7 CECAD Research Center, University of Cologne, Cologne, Germany

**Keywords:** Cancer, Metastasis, EMT, Non-coding RNA, Molecular mechanisms

## Abstract

Epithelial-mesenchymal transition (EMT) is a fundamental process for embryonic development during which epithelial cells acquire mesenchymal characteristics, and the underlying mechanisms confer malignant features to carcinoma cells such as dissemination throughout the organism and resistance to anticancer treatments. During the past decades, an entire class of molecules, called non-coding RNA (ncRNA), has been characterized as a key regulator of almost every cellular process, including EMT. Like protein-coding genes, ncRNAs can be deregulated in cancer, acting as oncogenes or tumor suppressors. The various forms of ncRNAs, including microRNAs, PIWI-interacting RNAs, small nucleolar RNAs, transfer RNA-derived RNA fragments, long non-coding RNAs, and circular RNAs can orchestrate the complex regulatory networks of EMT at multiple levels. Understanding the molecular mechanism underlying ncRNAs in EMT can provide fundamental insights into cancer metastasis and may lead to novel therapeutic approaches. In this review, we describe recent advances in the understanding of ncRNAs in EMT and provide an overview of recent ncRNA applications in the clinic.

## Background

Epithelial-mesenchymal transition (EMT) is a highly dynamic cellular process that transiently converts epithelial into mesenchymal characteristics. Although, EMT and the reverse process, mesenchymal-epithelial transition (MET), play crucial roles in specific steps of embryogenesis, the underlying molecular mechanisms are reactivated during tumor progression [1]. Upon activation of EMT, epithelial cells deconstruct their junctions, reprogram gene expression signatures and signaling pathways, modulate their cytoskeletal organization, destabilize apical–basal polarity and concomitantly acquire front–rear polarity [2]. These changes confer attributes of ‘high-grade malignancy’ on carcinoma cells, including motility, invasive behavior, cancer stem cell (CSC)-properties, resistance to conventional therapies and immunoevasive and immunosuppressive features [2]. Extracellular stimuli derived from the tumor microenvironment can trigger activation of several signaling pathways such as TGFβ/SMAD, WNT/β-catenin, Notch, and receptor tyrosine kinase signaling pathways [1]. These signaling pathways initiate EMT programs in tumor cells through induction of a core set of EMT transcription factors including three different protein families, namely the basic helix–loop–helix factors TWIST1 and TWIST2, the zinc-finger E-box-binding homeobox factors ZEB1 and ZEB2 and the SNAIL family of zinc-finger factors SNAI1 (also known as SNAIL) and SNAI2 (also known as SLUG) [3]. These transcription factors, in different combinations, repress the expression of epithelial-related genes such as *CDH1* (E-cadherin) and concomitantly induce the expression of mesenchymal-related genes such as *CDH2* (N-cadherin) and vimentin. Moreover, cellular motility and cytoskeletal changes are regulated by Rho GTPase family such as RhoA, RAC1 and CDC42 [1].

Preliminary investigation on the molecular mechanisms underlying EMT has widely focused on protein-coding genes, however, the discovery of non-coding RNAs (ncRNAs) has revolutionized our perception of the molecular mechanisms behind EMT. ncRNAs comprise a heterogeneous class of RNA transcripts (Table 1) with different biogenesis, length, and functions, which account for almost 60% of the human transcriptome [4–6]. Recently, functional studies have uncovered that ncRNAs, like protein-coding genes, can participate in diverse cellular processes such as EMT.

**Table 1:** General functions of non-coding RNAs

Abbreviation	Full name	Length (nt)	Function	
**Small non-coding RNA**				
miRNAs	microRNAs	19–24	lead to translational repression or degradation of the target mRNA	
piRNAs	Piwi-interacting RNAs	21–35	are loaded onto members of the PIWI subfamily of Argonaute proteins to repress transposons in germline cells	
snoRNAs	Small nucleolar RNAs	60–300	act as guide RNAs for the post-transcriptional modification of ribosomal and small nuclear RNAs	
tRFs	Transfer RNA-derived RNA fragments	14–30	like miRNAs, tRFs interact with Argonaute proteins to impair the translation of mRNAs through binding to target 3′ UTRs	
**Long non-coding RNA**				
NATs	Natural antisense transcripts	> 200	transcribed in the antisense direction of overlapping protein-coding genes	are involved in chromatin remodeling, transcriptional and post-transcriptional regulation, as well as translation and post-translational modifications
Pseudogenes	Pseudogenes	> 200	a subclass of the lncRNAs that resemble the protein-coding genes from which they are derived, but no longer produce functional proteins	
lincRNAs	long intergenic ncRNAs	> 200	transcribed from intergenic regions	
SNHGs	Small nucleolar RNA host genes	> 200	a type of lncRNA that contains both snoRNAs (which are produced from introns) and exons	
**Circular RNA**				
circRNAs	Circular RNAs	> 32	A type of covalently closed ncRNA that may interact with other molecules like miRNAs and proteins to regulate their functions	

In this review, we discuss the roles of various forms of ncRNAs in regulation of EMT in cancer and mention RNA modifications, which may result in the loss or gain of binding sites on ncRNAs with relation to EMT. Lastly, we focus on the potential clinical relevance of EMT-related ncRNAs in oncology.

## General functions of ncRNAs

### microRNAs

**Fig. 1:**
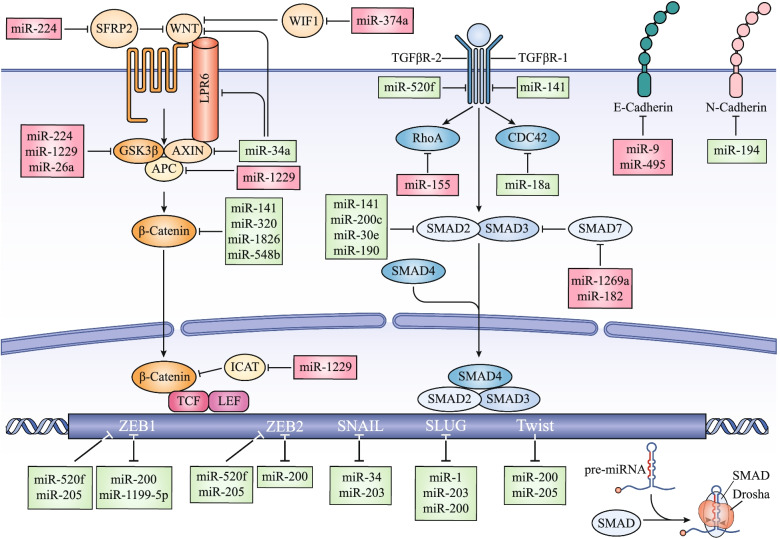
miRNAs regulate the EMT process in cancer. miRNAs post-transcriptionally suppress the expression of key players of the EMT program at multiple levels. Initially, EMT is triggered upon activation of several pathways such as TGF-β and WNT/β-catenin signaling pathways. The multiple components of these signaling pathways are targeted by various miRNAs. Activation of these signaling pathways promotes the expression of EMT-inducing transcription factors (ZEB, SNAIL and TWIST) that function pleiotropically to induce the acquisition of the mesenchymal properties. These transcription factors bind to the promoter regions of specific miRNAs and regulate their expression. On the other hand, miRNAs can target the 3′UTRs of the mRNAs that encode these transcription factors. Some of these miRNAs and transcription factors form a double negative feedback loop. The TGF-β signaling pathway regulates cytoskeletal dynamics through regulating RhoA and CDC42, which are targeted by miRNAs. mRNAs encoding adhesion molecules such as E-cadherin and N-cadherin are also targeted by miRNAs. Green boxes represent an EMT inhibitory role by the indicated miRNAs, whereas red boxes represent the induction of the EMT process by the indicated miRNAs.

microRNAs (miRNAs) constitute a highly conserved class of small ncRNAs (19–24 nucleotides) that lead to translational repression or degradation of target mRNAs through binding between the seed sequence of the miRNA and complementary sequences in the 3′ untranslated region (3′UTR) of the mRNA [7]. miRNAs can interact with various key players of EMT leading to the formation of highly complex gene-regulatory networks during tumor progression and metastasis (Fig. 1).

### PIWI-interacting RNAs

P-element Induced WImpy testis (PIWI)-interacting RNAs (piRNAs) are a class of small ncRNAs (21–35 nucleotides in length) that are loaded onto members of the PIWI subfamily of Argonaute proteins to repress transposons in germline cells [8]. However, recent findings have indicated that aberrantly expressed and malfunctioning piRNAs can lead to the development and progression of human malignancies [9, 10].

### Small nucleolar RNAs

Small nucleolar RNAs (snoRNAs) are generally classified as small ncRNAs of 60–300 nucleotides in length. snoRNAs act as guide RNAs for the post-transcriptional modification of ribosomal and small nuclear RNAs [11]. Based on structural characteristics, snoRNAs can be categorized into two families: C/D box snoRNAs (SNORDs) and H/ACA box snoRNAs (SNORAs) [12].

### Transfer RNA-derived RNA fragments

Transfer RNA-derived RNA fragments (tRFs) are single-stranded ncRNA transcripts (14–30 nucleotides) that are cleaved from mature transfer RNAs (tRNAs). Like miRNAs, tRFs interact with Argonaute proteins to impair the translation of mRNAs through binding to target 3′UTRs [13].

### Long non-coding RNAs

**Fig. 2:**
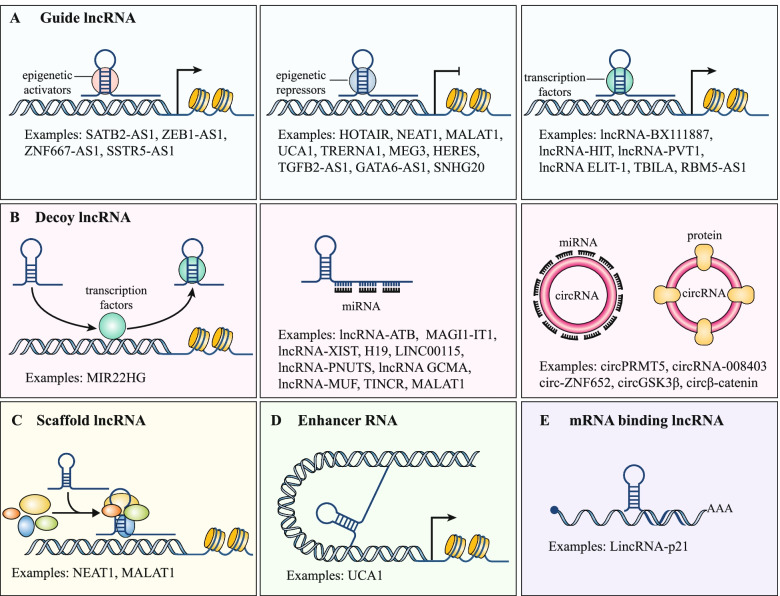
lncRNAs and circRNAs have versatile modes of action: **A** Guide lncRNAs can interact with regulatory proteins (such as epigenetic activators, epigenetic repressors or transcription factors) and direct them to their target regions. **B** Decoy lncRNAs and circRNAs can bind and sequester miRNAs or proteins. **C** Scaffold lncRNAs can function as a central platform to allow the assembly of various molecular components to facilitate their intermolecular interactions. **D** Enhancer RNAs are a class of ncRNAs that are transcribed from enhancer regions and act in regulating mRNA transcription. **E** mRNA-binding lncRNAs can bind to mRNAs and enhance or reduce its stability

Long non-coding RNAs (lncRNAs) constitute a heterogeneous class of ncRNAs that are at least 200 nucleotides [13]. Contrary to small ncRNAs, lncRNAs employ diverse mechanisms of action to perform their functional roles [14], and can be classified as scaffolds, decoys, or guides (Fig. 2). Scaffold lncRNAs can facilitate the interactions between various components of a complex, decoy lncRNAs can interact with either proteins or RNAs and titrate them away from their natural targets and guide lncRNAs can interact with proteins to localize them at specific genomic loci [12, 15] (Fig. 2). However, the miRNA decoy mechanism is somewhat controversial [16], in particular when the proposed decoy only contributes a tiny fraction of the total pool of miRNA targets in a cell [17]. Nevertheless, recent studies suggest that individual lncRNAs may function at substoichiometric ratios through seeding of concentration gradients in specific spatial territories or phase-separated compartments [18, 19] or by mediating target-directed miRNA degradation [20, 21].

### Circular RNAs

Circular RNAs (circRNAs) belong to a family of large ncRNAs that are characterized by covalently closed circular structures that are generated by a process called backsplicing of linear precursor RNAs [22, 23]. Like lncRNAs, circRNAs can act as decoys to sequester specific miRNAs [24, 25] or, conversely, may act to stabilize specific miRNAs [20, 22, 26]. Besides, circRNAs can interact with proteins by serving as scaffolds to assemble multiple components or as decoys that sequester the target proteins [13, 27–31]. In addition, some circRNAs have been proposed to encode unique proteins [32], however, the vast majority are believed to be non-coding [33]. As for the lncRNAs, controversies are associated with many circRNAs being proposed to function as miRNA decoys [34, 35]. Despite, hundreds of circRNAs have been claimed to have miRNA sponging properties, only few harbors more miRNA binding sites than expected by change [36] and most are very lowly expressed and probably non-functional [37]. Intriguingly, one of the most studied circRNAs in cancer, ciRS-7, was recently shown to be absent in cancer cells within solid tumors [38], even though numerous previous studies suggested that it functions as a miR-7 sponge in cancer cells. Rather it is expressed in the stromal cells and positive correlations with miR-7 target genes can be explained by stromal co-expression and were also observed for other stromal-enriched circRNAs that do not contain miR-7 binding sites [38].

## Small non-coding RNAs in EMT

### miRNA

#### miRNAs and EMT-transcription factors

Single miRNAs can target several genes and a single gene can be targeted by multiple miRNAs. Thus, in combination with downstream transcriptional changes arising from miRNA-targeted transcription factors highly complex gene-regulatory networks result, which might buffer or enforce signaling pathways [39]. For example, members of the miR-200 family and miR-205 cooperate to strongly inhibit EMT through suppressing ZEB1/2 expression [40]; conversely, ZEB1/2 directly suppress the transcription of the miR-200 family [41]. A similar mutually inhibitory loop exists between miR-1199-5p and Zeb1, coordinating EMT and tumor metastasis [42]. Such double-negative feedback loops might act as epithelial/mesenchymal switches that confer a high degree of plasticity required during the complex route from the primary tumor to metastasis [42, 43]. Similarly, double-negative feedback loops occur between SLUG and miR-1 and miR-200 [44], as well as between SLUG and miR-203. Interestingly, SLUG also inhibits miR-200 family members and thus might act in concert with the mutually inhibitory loop between miR-200 and ZEB1/2, to promote the EMT process [45]. Additionally, double-negative feedback loops also occurs between SNAIL and the miR-34 family (miR-34a/b/c) [46] as well as between SNAIL and miR-203 [47]. The miR-203/SNAIL regulatory complex in concert with the miR200/ZEB feedback loop might construct an EMT core network that could function as a robust switch regulating cell plasticity [47]. In addition, TWIST1 binds to the promoter regions of miR-200 and miR-205 and probably inhibits their expression to promote EMT in bladder cancer [48]. The detailed interplay between miRNAs and TWIST1 has been reviewed elsewhere [49]. Finally, the tumor-suppressor miRNA let-7 inhibits EMT through binding to multiple target sites in the 3′UTR of high mobility group A2 (HMGA2), a chromatin-binding protein that could directly bind to the promoter regions of *SNAIL* and *TWIST1* and promote their expression [50]. Together, several intertwined mutually inhibitory loops, in which miRNAs and EMT master transcription factors repress the expression of each other, robustly reinforce the epithelial or mesenchymal states and may orchestrate a quick and strong response to minimal stimuli [43].

#### miRNAs regulate cell adhesion and cytoskeletal components

In addition to targeting EMT master transcription factors, miRNAs also modulate the expression of cytoskeletal and cell adhesion components. For example, miR-9, which is directly activated by MYC and MYCN, induces EMT and metastasis by repressing the translation of E-cadherin [51]. Similarly, miR-495 represses E-cadherin expression and promotes EMT and tumorigenicity of breast cancer cells [52]. Alternatively, miR-194, whose expression is reduced in liver mesenchymal-like cancer cells, inhibits invasion and migration, partly through targeting of N-cadherin [53] and miR-30a, whose expression is induced by RUNX3, directly targets vimentin and represses its expression [54]. Finally, miR-375 suppresses the expression of CLAUDIN-1, thereby contributing to the dissolution of tight junctions during EMT in lung cancer [55].

#### miRNAs regulate EMT signaling pathways

The TGF-β signaling pathway, which plays a central role in promoting EMT in various tissue types, modulates miRNA expression at both transcriptional and post-transcriptional levels through Smads [56]. Upon treatment with TGF-β or bone morphogenetic protein (BMP), the Smad proteins directly bind to the RNA-Smad binding element (R-SBE) found in the stem region of primary transcripts of a set of miRNAs (such as miR-21and miR-181) and facilitate the recruitment of Drosha to the R-SBE-containing pri-miRNAs, promoting the processing of these primary transcripts [57, 58]. Moreover, TGF-β control the transcription of various miRNAs through R-SBEs in the miRNA promoter. For example, TGF-β released from M2 macrophages promotes binding of Smad2/3 to the miR-362-3p promoter, resulting in upregulation of miR-362-3p in hepatocellular carcinoma (HCC) cells [59]. miR-362-3p directly targets CD82, a key player in the tetraspanin network, thereby maintaining EMT in HCC cells [59]. In addition, TGF-β-activated Smads indirectly regulate miRNA expression through activation of transcription factors that bind to their promoters [56]. On the other hand, miRNAs may regulate the TGF-β signaling pathway at multiple levels through targeting of ligands and receptors as well as Smad and non-Smad pathway components. For instance, members of the miR-200 and/or miR-30 families inhibit the invasive ability and promote MET in anaplastic thyroid carcinoma cells through targeting TGFBR-1 and Smad2 [60]. Similarly, miR-190, which is directly suppressed by ZEB1, inhibits TGF-β-induced EMT and metastasis through targeting Smad2 in breast cancer both in vitro and in vivo [61].

Similarly, WNT/β-catenin signaling orchestrates a reciprocal crosstalk with miRNAs to regulate EMT in tumor cells. The β-catenin/LEF1 complex transcribes expression of miR-150, which in turn directly targets CREB1 and EP300 to facilitate EMT in colorectal cancer (CRC) cells [62]. On the other hand, the miR-34 family, inhibits EMT through targeting key components of the canonical WNT signaling pathway such as β-catenin, WNT1, WNT3, LRP6, SNAIL, and AXIN2 [63, 64]. Moreover, miR-374a, whose ectopic expression induces EMT and metastasis both in vitro and in vivo, promotes the WNT/β-catenin pathway by targeting negative regulators of the WNT/β-catenin signaling cascade, such as WIF1, PTEN, and WNT5A [65].

#### miRNAs regulate cell motility

miRNAs can modulate actin cytoskeletal dynamics during EMT. A study using high-throughput sequencing of RNA isolated by HITS-CLIP technology uncovered hundreds of miR-200a and miR-200b targets that are predominantly enriched for genes associated with the cytoskeletal remodeling [66]. Similarly, miR-23b, a metastatic suppressor miRNA, could regulate cytoskeletal remodeling in breast cancer through directly suppressing a set of genes, including *PAK2*, *LIMK2*, *ARHGEF6*, *CFL2*, *PIK3R3*, *PLAU*, and *ANXA2* [67]. Inhibition of miR-23b, using a miR-23b sponge construct, induces spontaneous metastasis in an orthotopic mouse model of breast cancer [67]. In contrast, miR-155 induces EMT, tight junction dissolution, migration and invasion through targeting RhoA in breast cancer [68].

### Other small non-coding RNAs and EMT

In addition to miRNAs, other small non-coding RNAs, such as snoRNAs, piRNAs and tRFs can regulate EMT in cancer; however, their contribution to EMT, is only beginning to be uncovered. For instance, SNORD78, which is upregulated in non-small cell lung cancer (NSCLC) and associated with poor prognosis, may contribute to the invasion and EMT in NCSLC probably through aberrant methylation of *CDH1* [69] and SNORD113–1, which is downregulated in HCC, inhibits tumor cell growth via inhibiting the phosphorylation of ERK1/2 and Smad2/3 in the MAPK-ERK and TGF-β pathways [70].

piRNAs may also regulate EMT. piR-932 is highly expressed in breast cancer cells with EMT and CSC phenotypes [9]. Functionally, the combination of piR-932 and PIWIL2 serve as a positive regulator of EMT in breast CSCs through mediating the methylation of *LXN* [9]. Similarly, piR-1037, which is upregulated in oral squamous cell carcinoma (OSCC) cells and xenografts, promotes EMT and motility of OSCC cells likely through interacting with XIAP [10].

Besides, tRF may participate in the regulation of EMT. For example, tRF/miR-1280, a small fragment (17-bp) that is derived from both tRNA^Leu^ and pre-miRNA inhibits CSC and EMT phenotypes and metastasis in CRC [71]. Mechanistically, tRF/miR-1280 interacts with 3′UTR of *JAG2*, a Notch ligand, and inhibits its expression, leading to suppression of Notch/Gata and miR-200b pathways [71].

## Long non-coding RNAs in EMT

### lncRNAs

#### lncRNAs and EMT-transcription factors

Recently, an increasing number of lncRNAs have been implicated in EMT and tumor metastasis through regulating EMT-transcription factors via various mechanisms. For instance, the lncRNA antisense to *ZEB1* (*ZEB1-AS1*) can interact with MLL1 and recruit it to the promoter region of *ZEB1* to epigenetically induce *ZEB1* transcription, promoting EMT and tumor metastasis [72, 73]. In addition, lncRNA-BX111887 directly interacts with transcriptional factor Y-box protein (YB1) and recruits it to the *ZEB1* promoter region and subsequently transactivates *ZEB1* expression, promoting tumor growth and metastasis of pancreatic cancer in a xenograft mouse model [74]. Besides, a set of lncRNAs has been identified that can post-transcriptionally regulate ZEB expression by sequestering miRNAs (Table 2). lncRNA-activated by TGF-β (lncRNA-ATB) functions as a competitive endogenous RNA (ceRNA) to sequester members of the miR-200 family and sustain ZEB1/2 expression in HCC [75]. Interestingly, SNHG14, through acting as a miRNA sponge for miR-5590-3p, derepresses the expression of *ZEB1* which in turn induces SNHG14 expression, forming a positive feedback loop [76]. Moreover, lncRNA RP11–138 J23.1 (RP11) binds hnRNPA2B1 protein to facilitate the interaction between hnRNPA2B1 and the mRNA of *SIAH1* and *FBXO45*, ubiquitin E3 ligases that stimulate ZEB1 degradation. This complex facilitates the mRNA degradation of Siah1 and Fbxo45, preventing ZEB1 degradation [77]. Another lncRNA, lncRNA-HIT, interacts with ZEB1 and enhances its protein stability [78].

**Table 2:** EMT-related long non-coding RNAs

lncRNAs	Mode of action	EMT component	References
**Related to EMT-transcription factors**			
lncRNA-ATB, MAGI1-IT1, lncRNA-XIST, LINC00115, H19, lncRNA-PNUTS, lncRNA-PTAR, ZFAS1, OIP5-AS1, SNHG14, SNHG16	Sponging miRNA	ZEB1/2	[75, 76, 79–88]
lncRNA RP11–138 J23.1	Post-translationally		[77]
ZEB1-AS1	Epigenetically		[72, 89]
lncRNA-BX111887	Transcriptionally		[74]
lncRNA PVT1	Transcriptionally	SLUG	[90]
AC026904.1	Enhancer RNA		[91]
lncRNA GCMA, lncRNA TINCR, UCA1,	Sponging miRNA		[91–93]
SNHG15	Post-translationally		[94]
lncRNA GCMA, lncRNA-MUF, SNHG7	Sponging miRNA	SNAIL	[92, 95, 96]
SATB2-AS1	Epigenetically		[97]
lncRNA JPX, LINC01296	Sponging miRNA	TWIST1	[98, 99]
**Related to cell adhesion and cytoskeletal components**			
MALAT1, UCA1, TRERNA1, MEG3, ZNF667-AS1, SSTR5-AS1, SNHG20	Epigenetically	E-cadherin	[100–106]
MEG3,	Sponging miRNA		[107, 108]
NEAT1	Transcriptionally	ZO1	[109]
MALAT1, H19	Sponging miRNA	vimentin	[79, 110]
LINC00675, FTX, AOC4P	Post-translationally		[111–113]
**Related to EMT signaling pathways**			
lncRNA-ATB, lncRNA-XIST, LINC01278, OIP5-AS1	Sponging miRNA	TGF-β pathway components	[114–117]
MIR22HG	Protein interaction		[118]
TGFB2-AS1	Epigenetically		[119]
MALAT1	Post-translationally		[120]
lncRNA HERES, NEAT1, GATA6-AS1	Epigenetically	WNT pathway components	[121–123]
MIR100HG, lincRNA-p21,	Post-transcriptionally		[124, 125]
CYTOR, lncRNA-MUF, LncCCAT1	Post-translationally		[95, 126, 127]
LncCCAT1, SNHG5, SNHG6	Sponging miRNA		[127–129]
**Related to cell motility**			
lncRNA-UCA1	Sponging miRNA	FSCN1	[130]
lncMER52A	Post-translationally	p120-catenin	[131]
LCAT1, H19, MALAT1, NORAD, XIST, CTC-497E21.4, TP73-AS1, AURKAPS1, FTH1P3	Sponging miRNA	Rho GTPase components	[132–140]

Similarly, the expression of SNAIL and SLUG is regulated by lncRNAs through multiple mechanisms. The lncRNA PVT1 transcriptionally promotes SLUG expression through directly interacting with the transcription factor STAT3 and recruiting it to the *SNAI2* promoter [90]. Interestingly, the PVT1 locus produces multiple linear and circular transcripts, and it is important to consider that not all molecular assays can distinguish the linear and circular forms [141]. Besides, multiple lncRNAs have been identified that could post-transcriptionally regulate SNAIL or SLUG expression (Table 2). For instance, lncRNA GCMA functions as a ceRNA for miR-34a and miR-124 to derepress SNAIL and SLUG, respectively, thereby promoting EMT in vitro and metastasis in vivo [92]. Interestingly, TGF-β-induced lincRNAs, AC026904.1 and UCA1, cooperatively promote SLUG expression at transcriptional and post-transcriptional levels, respectively; UCA1 functions as a ceRNA to sequester miR-1 and miR-203 and derepress SLUG expression, while, AC026904.1 behaves as an enhancer RNA and directly binds the promoter region of *SNAI2* to facilitate its transcription [91]. Besides, lncRNAs, such as HOTAIR and NEAT1, can mediate interaction between SNAIL and epigenetic machineries to specifically localize this complex at SNAIL binding sites on epithelial gene promoters thereby inducing EMT [142, 143]. Moreover, SNHG15 interacts with SLUG and prevents BTRC-induced SLUG ubiquitination and degradation, promoting EMT in CRC [94].

Finally, lncRNA JPX derepresses *TWIST1* expression through sponging miR-33a-5p and this regulatory axis activates EMT and metastasis in vitro and in vivo through inducing WNT/β-catenin signaling [98]. Additionally, TWIST1 transcriptionally induces lncRNA LINC01296, which sequesters miR-598 and in turn derepresses its direct target TWIST1, thereby forming a positive feedback loop [99].

#### lncRNAs regulate cell adhesion and cytoskeletal components

Several lncRNAs (listed in Table 2) have been demonstrated to interact with epigenetic regulators to recruit them to the regulatory regions of *CDH1*. For example, TRERNA1 behaves as a scaffold to facilitate the recruitment of EZH2 to the *CDH1* promoter and silence its expression in gastric cancer [100]. Additionally, MEG3 induces E-cadherin expression via functioning as a ceRNA for miR-21 and miR-421 [107, 108]. Besides, NEAT1 facilitates the interaction between FOXN3 and SIN3A, a transcriptional repressor complex that repress the expression of GATA3 and ZO1 (an epithelial marker), resulting in EMT promotion [109]. In addition to epithelial markers, mesenchymal markers such as vimentin are regulated by lncRNAs. As previously indicated, LINC00460 directly interacts with PRDX1 and recruits it to the promoter region of vimentin and other mesenchymal markers, and consequently induces their transcription [144]. In addition, MALAT1 and H19 upregulate vimentin expression through acting as a sponge for miR-30a-5p and miR-138, respectively [79, 110]. lncRNAs may also post-translationally regulate vimentin stability [111–113]. For instance, amine oxidase, copper containing 4, pseudogene (*AOC4P*) interacts with vimentin to promote its ubiquitin-dependent degradation, impairing EMT, tumor growth and metastasis in animal models [111] and LINC00675 interacts with vimentin to facilitate its phosphorylation thereby destabilizing vimentin filaments and inhibiting metastasis in gastric cancer [112].

#### lncRNAs regulate EMT signaling pathways

lncRNAs can regulate key components of TGF-β pathway through various mechanisms. TGFB2-antisense RNA1 (TGFB2-AS1), which is induced by canonical TGF-β signaling, physically associates with the PRC2 adaptor protein EED, and recruits it to the TGF-β-target genes to facilitate their methylation and suppression, thus forming a feedback loop [119]. Moreover, lncRNA ELIT-1, which its upregulation positively correlates with poor outcome in patients with lung adenocarcinoma and gastric cancer, interacts with Smad3 and recruits it to the promoters of multiple genes including SNAIL, vimentin, N-cadherin, API-1, and ELIT-1 itself to accelerate their transcription, forming a positive feedback loop which promotes EMT [145]. Additionally, lncRNA-ATB and lncRNA-XIST could promote TGFβ2 expression and tumor progression through acting as a sponge for miR-141-3p [114, 115]. Furthermore, MIR22HG lncRNA inhibits TGF-β signaling and EMT via competitively binding to Smad2 and perturbing the interaction between Smad2 and Smad4 in CRC [118]. Moreover, MALAT1 cooperates with SETD2 to form a scaffold, which facilitates binding of phosphorylated Smad2/3 to their specific phosphatase PPM1A, thus promoting the dephosphorylation of Smads and subsequently inhibiting TGF-β signaling [120].

Besides, lncRNAs can regulate various key players of the WNT pathway. For example, GATA6 antisense RNA 1 (GATA6-AS1) interacts with EZH2 and increases its occupation at *FZD4* promoter to repress its expression, leading to inactivation of WNT/β-catenin pathway [121]. Similarly, oncogenic lncRNAs such as HERES and NEAT1 can epigenetically activate the WNT/β-catenin pathway via binding to EZH2 and recruiting it to the promoter regions of multiple negative regulators of the WNT/β-catenin pathway [122, 123]. Furthermore, RBM5 antisense (RBM5-AS1) directly associates with β-catenin and facilitates the recruitment of β-catenin and TCF4 complex to the WNT target genes *SGK1*, *YAP1*, and *MYC*, activating the WNT pathway in CRC [146]. Moreover, lincRNA-p21 physically associates with mRNAs encoding β-catenin (CTNNB1) and JunB (JUNB) and induces their translational repression via translational repressor Rck, leading to inhibition of WNT/β-catenin pathway [124]. Besides, lncRNA cytoskeleton regulator RNA (CYTOR) interacts with cytoplasmic β-catenin to prevent casein kinase 1 (CK1)-mediated phosphorylation of β-catenin, which leads to accumulation of β-catenin in the nucleus and induces transcriptional activity of the β-catenin/TCF complex [126]. Interestingly, lncRNA-MUF and LncCCAT1 facilitate the interaction between ANXA2 and GSK3β, which prevents β-catenin phosphorylation and degradation, through disrupting the formation of the GSK3β/β-catenin complex [95, 127]. In addition, NEAT1 directly interacts with DDX1, a protein that binds to β-catenin and enhances its protein stability and transcriptional activity, thus activating the WNT/β-catenin pathway [147].

#### lncRNAs regulate cell motility

lncRNAs also regulate EMT through modulating cytoskeletal dynamics. For instance, lncRNA-UCA1 promotes EMT in bladder cancer through sequestering tumor suppressive miR-145 that targets ZEB1/2 and fascin homologue 1 (FSCN1), an actin-binding protein that regulate actin-based cellular protrusions and cellular motility [130]. Besides, lncMER52A directly interacts with and stabilizes p120-catenin by preventing β-TrCP1-mediated ubiquitination and degradation [131]. Moreover, *ABHD11* antisense RNA1 (ABHD11-AS1) and actin filament-associated protein 1-antisense RNA1 (AFAP1-AS1) directly interact with RhoC and facilitate EMT in ovarian cancer and osteosarcoma, respectively [148, 149]. Moreover, multiple, lncRNAs have been shown to upregulate the expression of Cdc42, RAC1 or RhoA through functioning as decoys for various miRNAs (Table 2), including H19 and MALAT1 that upregulate Cdc42 by sponging miR-15b and miR-1, respectively [132, 133].

### circRNAs

#### circRNAs and EMT-transcription factors

In the first study to indicate a role for circRNAs in EMT a high-throughput RNA sequencing analysis demonstrated that the expression of hundreds of circRNAs are modulated during EMT, most of them upregulated [150]. The functions of circRNAs in EMT remain largely unknown, although more recently, several circRNAs have been proposed to regulate EMT transcription factors. For example, circPRMT5 may induce EMT in UCB cells and promotes an aggressive phenotype in a xenograft mouse model via sponging miR-30c, a tumor suppressor that directly inhibits SNAIL expression [151]. Similarly, circRNA_0084043 and circ-ZNF652 was suggested to derepress SNAIL expression through sponging of miRNAs [152, 153] and has-circ-000684, which is upregulated in gastric cancer cell lines and tumor tissues, derepresses ZEB1 by sponging miR-186 [154]. Moreover, circPCNXL2, which is highly expressed in clear cell renal cell carcinoma (ccRCC) and positively correlates with poor prognosis, induces invasion and proliferation in vitro and promotes tumor growth in a xenograft mouse model by acting as a miRNA sponge to inhibit the miR-153-mediated suppression of ZEB2 [155]. In addition, EMT transcription factors may transcriptionally regulate circRNA expression. For example, TWIST1 binds to the promoter region of the *CUL2* gene to selectively induce transcription of a circRNA (circRNA-10,720), which exerts its oncogenic function in HCC by sponging multiple miRNAs that target vimentin [156].

#### circRNAs regulate cell junction and cytoskeletal components

Several circRNAs have been identified as EMT regulators through modulating key components of cell junctions and the cytoskeleton. Circ-AKT3 may sponge miR-296-3p to promote *CDH1* expression, thereby suppressing migration and invasion in vitro as well as tumor cell dissemination in vivo [157]. Similarly, circPTPRA regulates E-cadherin and EMT in NSCLC by sponging of miR-96-5p, permitting the expression of the downstream tumor suppressor RASSF8, which interacts with E-cadherin and stabilizes adhesive junctions [158, 159]. In addition, circAMOTL1L upregulates the expression of protocadherin-α (Pcdha), a member of cadherin superfamily which mediates cell-cell adhesion, through behaving as a miRNA sponge for miR-193a-5p, thus, its loss in prostate cancer (PCa) promotes cell migration, invasion and EMT in vitro and tumor growth in vivo [160]. Interestingly, circPTK2 can bind to Ser38, Ser55, and Ser82 sites of vimentin and induce EMT in vitro and in vivo and targeting of circPTK2 using shRNA significantly suppressed tumor metastasis in a xenograft mouse model of CRC [161].

#### circRNAs regulate EMT signaling pathways

Deregulated circRNAs in cancer may also regulate EMT-related signaling pathways such as the TGF-β and WNT pathways. For example, circPTK2 was suggested to sponge miR-429 and miR-200b-3p to derepress TIF1γ, a protein that negatively regulates TGF-β/Smad signaling through Smad4 ubiquitination and competing with Smad4 to bind Smad2/3 complex [162–164]. Additionally, circANKS1B, whose upregulation is associated with lymph node metastasis and poor prognosis, induces EMT through sponging multiple miRNAs, including miR-148a and miR-152-3p that target USF1, a transcription factor that induces expression of TGF-β and ESPR1, forming a feedback loop [165]. Similarly, circUHRF1 promotes EMT in vitro and tumor growth in vivo via sponging miR-526b-5p to derepress the expression of c-Myc, which in turn promotes expression of TGF-β1 and ESRP1 [166]. Additionally, hsa_circ_0009361, which is downregulated in CRC tissues and cells, inhibits Wnt/β-catenin pathway and EMT through sponging miR-582 to upregulate APC2, a Wnt/β-catenin pathway inhibitor [167].

Another interesting study suggests that circGSK3β, directly interacts with GSK3β and inhibits GSK3β activity, thereby protecting β-catenin from phosphorylation and degradation which subsequently results in promoting β-catenin pathway and EMT in esophageal squamous cell carcinoma (ESCC) [168]. GSK3β-mediated β-catenin phosphorylation and degradation can be also antagonized via direct interaction of GSK3β with a novel 370-amino acid β-catenin isoform that is derived from circβ-catenin [169]. Moreover, circ-CTNNB1 directly interacts with DEAD-box polypeptide 3 (DDX3) to promote its physical association with transcription factor Yin Yang 1 (YY1), leading to transactivation of *YY1* and transcriptional regulation of β-catenin pathway key components such as *WNT1*, *WNT3*, *AXIN2*, *FZD10* and *BMP4* [170]. Besides, circRNA-MYLK, whose expression is upregulated in bladder cancer and positively associated with advanced clinical stage, behaves as a miR-29a sponge to upregulate VEGF expression inducing VEGFA/VEGFR2 signaling pathway which consequently promotes EMT and angiogenesis in bladder cancer [171].

#### circRNAs regulate cell motility

circRNAs may also regulate key players of cytoskeletal dynamics. Interestingly, circHIAT1 was suggested to behave as a ‘miRNA reservoir’ to stabilize multiple tumor suppressive miRNAs such as miR-195-5p/29a-3p/29c-3p that target CDC42 in ccRCC [172]. Additionally, FLI1 exonic circRNAs (FECR) including FECR1 and FECR2 participate in the activation of ROCK1, a key effector of RhoA GTPase that promotes actin polymerization, through sequestering miR-584-3p from binding to ROCK1 [173]. Furthermore, circ-133, which is enriched in the exosomes derived from hypoxic CRC cells promotes tumor metastasis through regulating miR-133a/GEF-H1/RhoA axis [174]. These data indicate that the deregulated circRNA could regulate the expression of major regulators of cytoskeletal dynamics.

## RNA modifications in EMT

### Adenosine-to-inosine (A-to-I) RNA editing modifies targeting by ncRNAs

**Fig. 3:**
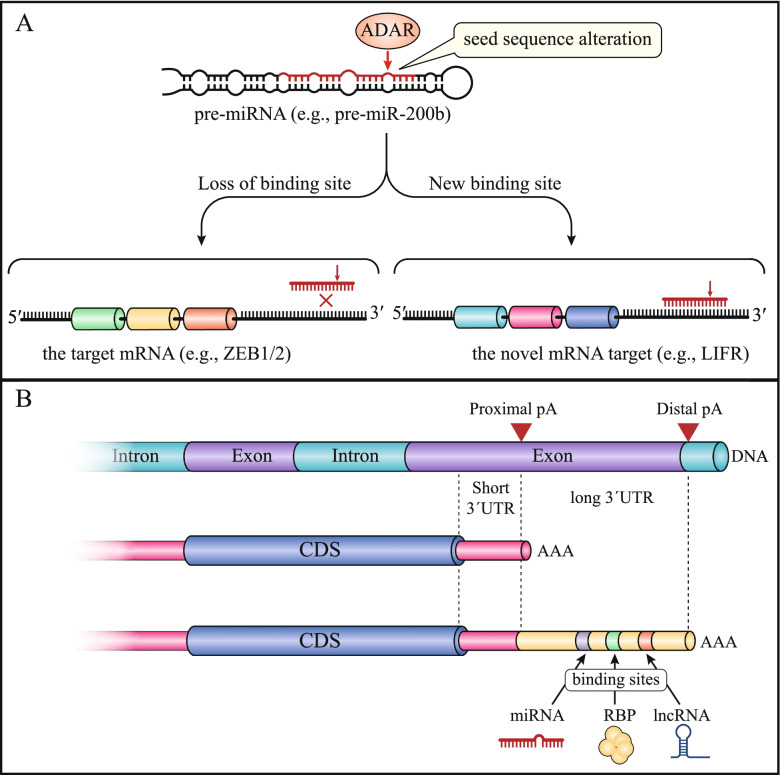
RNA modification alters ncRNA targeting. **A** Adenosine-to-inosine (A-to-I) editing of the seed sequence of a miRNA can alter the base pairing properties of the miRNA. The double-stranded RNA-specific adenosine deaminases (ADARs) can interact with target site (here the target site is the seed sequence of miR-200b) and change adenosine bases to inosine, thereby changing the sequence of the target site. In this example, ADARs change the seed sequence of the miR-200b. The edited miR-200b loses its ability to interact with 3′UTR of *ZEB1* and *ZEB2*; while it concomitantly acquires the capability to interact with novel targets such as *LIFR*, a well-known anti-metastatic gene. Therefore, this process can change the tumor-suppressive miR-200b to an oncogenic miRNA. **B** Alternative polyadenylation (APA) in the 3′UTR can generate multiple mRNA transcripts with different 3′ UTRs. As shown here, the 3′ UTR of the candidate gene includes two APA sites which can give rise to two isoforms with short and long 3′ UTRs. The short isoform might produce more proteins due to escaping from repression by various components such as miRNAs, lncRNAs, and RNA-binding proteins

RNA editing is a post-transcriptional modification which remodels the transcriptional landscape and consequently governs cell fate decisions. To date, more than 100 distinct modifications of RNA have been identified, emphasizing the critical role of these modifications on genome output [175]. Of the RNA modifications, the conversion of adenosine to inosine, termed A-to-I editing, is the most abundant form of RNA editing in Metazoans which is mediated by the adenosine deaminase acting on RNA (ADAR) family of enzymes [175]. Upon A-to-I editing inosines are recognized as guanosines and base-pair with cytosines, suggesting that A-to-I editing can modify the transcripts secondary structures [176]. A-to-I editing can occur in protein-coding sequences, however most of these events occur in non-coding regions [177, 178]. RNA editing in miRNAs may have a profound effect on miRNA regulation, because alteration of a single nucleotide in the seed sequence of an individual miRNA can alter the base pairing properties, potentially leading to creation or disruption of miRNA binding sites [6]. For example, miR-200b has been found to be overedited in various tumors, and its edited form correlates with worse prognosis [179, 180]. Unlike wild-type miR-200b, the edited miR-200b loses its capacity to suppress *ZEB1* and *ZEB2* and concomitantly acquires the capability to suppress novel targets such as *LIFR*, a well-known anti-metastatic gene (Fig. 3A) [179]. This editing is mediated by ADAR1; thus, its inhibition impairs miR-200b editing which in turn inhibits tumor cell invasion and migration and impairs tumor growth in a xenograft mouse model of thyroid cancer [179, 180]. ADAR1 can also interact with Dicer and facilitates maturation of a set of oncogenic miRNAs that promotes migration, invasion and EMT in OSCC [181], and has recently been shown to regulate a large number of cancer-relevant circRNAs via both editing-dependent and independent mechanisms [182].

### Alternative polyadenylation modifies targeting by ncRNAs

High-throughput sequencing technologies, genome-wide experimental and bioinformatic tools have uncovered that more than 70% of mammalian genes have alternative polyadenylation regions in the 3ʹUTR, which can give rise to multiple mRNA transcripts with different 3′ UTRs [183]. Tumor cells mainly express mRNA isoforms with shortened 3′UTRs which results from alternative polyadenylation [184]. Shortening of 3′UTRs can remarkably generate more protein relative to their full-length 3′ UTR counterparts probably by escaping from miRNA-mediated translational repression (Fig. 3B) [184]. Tumor cells expressing mRNA isoforms with shorter 3′UTR are considered to be more aggressive and result in a poorer prognosis [185]. In pancreatic ductal adenocarcinoma (PDAC), treatment with genotoxic agents induces shortening of *ZEB1* 3′UTR which increases ZEB1 protein production through escaping from repression by miRNAs such as miR-200 [186]. ZEB1 protein production directly associates with alternative polyadenylation of *ZEB1* 3′ UTR [186]. Additionally, during EMT and tumor progression, the *TWIST1* 3′UTR, containing several regulatory elements, is shortened which leads to increased TWIST1 protein production [187]. Similarly, the 3′ UTR of *RAC1* and Fibronectin type III domain containing 3B *(FNDC3B*) is shortened which results in enhanced protein production during cancer progression [188, 189]. FNDC3B directly associates with and stabilize myosin heavy chain 9 (MYH9) to facilitate Wnt/β-catenin pathway activation [189].

## EMT-related ncRNAs as cancer biomarkers

Accumulating evidence have demonstrated that ncRNAs are dysregulated across diverse cancers. Given the tissue and stage-specific expression patterns of many ncRNAs they are promising biomarkers for cancer detection and prognosis (Table 3). Owing to the covalently closed structure, circRNAs are highly resistant to exonucleases, making them a promising biomarker compared to other sub-classes of ncRNAs as they may be more readily detected in serum, plasma or urine, where the obtaining procedures are relatively convenient and less invasive compared to obtaining a tumor biopsy [190–192]. For example, the plasma levels of circGSK3β, a circRNA that promotes EMT and cancer progression, were shown to be significantly elevated in patients with ESCC [168]. Additionally, circPTK2 which promotes EMT and tumor metastasis, is significantly elevated in the serum of patients with CRC and the expression levels of circPTK2 is a metastatic indicator in patients with CRC [161]. Intriguingly, ncRNAs can be selectively packaged into exosomes or other extracellular vesicles, which protect them from RNase-mediated degradation in the extracellular space and in body fluids. The serum exosomal FECR1 (FLI1 exonic circular RNA) was shown to be markedly higher in patients with SCLC compared with that in heathy individuals and higher levels of exosomal FECR1 could serve as a biomarker of an unfavorable outcome in patients with SCLC [173]. Moreover, circPRMT5 is enriched in the serum and urinary exosomes of patients with UCB and upregulation of circPRMT5 in the serum and urinary exosomes is associated with lymph node metastasis [151]. These findings suggest that EMT-related ncRNAs, particularly circRNAs, can serve as diagnostic or prognostic biomarkers in cancer.

**Table 3:** Selected list of clinical trials exploring ncRNAs as cancer biomarkers

Name	ncRNA Class	Cancer Type	Source	Implications	Trial Identifier	Trail Status
let-7	miRNA	Non-Hodgkin’s Lymphoma and Acute Leukemia	Tissue	Diagnostic	NCT05477667	Recruiting
miR-10b	miRNA	Glioma	Tissue, Blood and Cerebrospinal Fluid	Prognostic for OS and PFS	NCT01849952	Recruiting
miR-30	miRNA	Prostate Cancer	Blood	Diagnostic and Prognostic	NCT04662996	Recruiting
miR-31-3p	miRNA	Colorectal Cancer	tissue	Prognostic for DFS, OS and SAR	NCT03362684	Completed
miR-34a	miRNA	Acute Myeloid Leukemia	Tissue	Diagnostic	NCT01057199	Completed
miR-141	miRNA	Prostate Cancer	Tissue	Prognostic for PFS	NCT02391051	Recruiting
					NCT04283032	Unknown
miR-200(a, b, c)	miRNA	Ovarian Cancer	Blood	Prognostic for PFS	NCT02758652	Recruiting
					NCT04283032	Unknown
miR-200b	miRNA	Ovarian, and Colon Cancer (Stage IV)	Blood	Prognostic for PFS	NCT03776630	Recruiting
				Predictive and Prognostic.	NCT04149613	Recruiting
miR-203	miRNA	Colon Cancer (Stage IV)	Blood	Predictive and Prognostic.	NCT04149613	Recruiting
miR-374a	miRNA	Prostate Cancer	Blood	Prognostic for PFS	NCT05022914	Recruiting
miR-375	miRNA	Prostate Cancer, Merkel Carcinoma	Tissue, Blood	Diagnostic	NCT04283032	Unknown
					NCT04705389	Unknown
CCAT1	lncRNA	Colorectal Cancer	Blood	Diagnostic	NCT04269746	Unknown
H19	lncRNA	Liver Cancer	Blood	Diagnostic	NCT04767750	Completed
HOTAIR	lncRNA	Thyroid Cancer	Blood	Diagnostic	NCT03469544	Unknown
PVT1	lncRNA	Gastrointestinal Cancer	Blood	Diagnostic	NCT03076502	Unknown
UCA-1	lncRNA	Liver Cancer	Tissue	Diagnostic	NCT05088811	Recruiting
XIST	lncRNA	Acute Myeloid Leukemia	Tissue, Blood	Diagnostic	NCT04288739	Not Yet Recruiting

## EMT-related ncRNAs as therapeutic targets

The EMT program leads to increased invasion and migration of tumor cells. Thus, targeting of EMT-related ncRNAs may hold therapeutic potential. To date, miRNAs are the most widely studied class of ncRNAs in cancer. One of the features of miRNAs that makes them attractive tools and targets for novel therapeutic strategies is their capacity to regulate several targets within a specific pathway or a set of targets across various pathways. Thus, certain miRNAs may lead to a stronger therapeutic effect if their multiple targets are enriched within a specific pathway [39]. The miR-200 family that regulates EMT process at multiple levels through targeting a set of mRNAs involved in Rho signaling pathway, invadopodia formation, focal adhesions, and EMT-related transcription factors, is a prominent example [39, 66].

The current strategies for miRNA-based therapeutics include reintroduction of tumor suppressive miRNAs using synthetic double-stranded oligoribonucleotides (also known as miRNA mimics) and/or inhibition of the oncogenic miRNAs via single-stranded antisense oligoribonucleotides (also known as antimiRs). These oligoribonucleotides are chemically modified to prevent RNase-mediated degradation, enhance binding affinity, and improve their pharmacokinetic characteristics in vivo [193, 194]. In addition, various delivery vehicles, including lipid-based nanoparticles and peptide and polymer-based systems are developed to encapsulate oligoribonucleotides to protect them from degradation and facilitate endosomal escape [194].

To date, several miRNAs have reached clinical development (Table 4). For example, miR-16 mimics has recently completed a phase I clinical trial (NCT02369198) with encouraging results in patients with malignant pleural mesothelioma [195]. Additionally, a locked nucleic acid (LNA)-modified antimir-155 has advanced to a phase II clinical trial (NCT03837457) in patients with certain lymphomas and leukemias.

**Table 4:** Selected list of ncRNAs as potential therapeutic targets

Name (Therapeutic agent)	ncRNA Class	Cancer Type	Delivery system	Developmental stage	References
MesomiR 1 (miR-16 mimics)	miRNA	Malignant Pleural Mesothelioma, Non-Small Cell Lung Cancer	Non-living bacterial minicells	Phase I (NCT02369198), Completed	[195]
Cobomarsen /MRG-106 (anti–miR-155)	miRNA	Cutaneous T-Cell Lymphoma/Mycosis Fungoides	LNA-modified antisense inhibitor	Phase II (NCT03713320), Terminated	[196]
MRX34 (miR-34 mimic)	miRNA	Multiple Solid Tumors	Lipid nanoparticles	Phase I (NCT01829971), Terminated	[197]
miR-34 and let-7 mimics	miRNA	Non–Small Cell Lung Cancer	Encapsulated in neutral lipid emulsion	Pre-clinical (Transgenic)	[198]
miR-199a/b-3p mimics and antimiR-10b	miRNA	Hepatocellular Carcinoma	Polymer-based nanoplatform	Pre-clinical (xenograft and patient-derived xenograft)	[199]
antagopiR54265	piRNA	Colorectal Adenocarcinoma	2ʹ-O-methoxyethy modified and 5′-cholesterol-conjugated piRNA inhibitor	Pre-clinical (xenograft)	[200]
anti-SNORA23	snoRNA	Pancreatic Ductal Adenocarcinoma	Antisense oligonucleotide	Pre-clinical (xenograft)	[201]
anti-Leu3′tsLNA	tsRNA	Hepatocellular Carcinoma	LNA-modified antisense inhibitor	Pre-clinical (patient-derived xenograft)	[202]
ARLNC1	lncRNA	Prostate Cancer	Antisense oligonucleotide	Pre-clinical (xenograft)	[203]
LINK-A	lncRNA	Breast Cancer	LNA-modified antisense inhibitor	Transgenic	[204]
SAMMSON	lncRNA	Melanoma	GapmeR	Pre-clinical (patient-derived xenograft)	[205]
MALAT1	lncRNA	Lung And Breast Cancer	Antisense oligonucleotide	Pre-clinical (xenograft and Transgenic)	[206–208]
circ-133	circRNA	Colorectal Cancer	Exosome-mediated delivery	Pre-clinical (xenograft)	[174]
circPTK2	circRNA	Colorectal Cancer	Lentivirus	Pre-clinical (patient-derived xenograft)	[161]
circAGO2	circRNA	Gastric Adenocarcinoma	Lentivirus	Pre-clinical (xenograft)	[209]
CircLONP2	circRNA	Colorectal Cancer	Antisense oligonucleotide	Pre-clinical (xenograft)	[210]

The development of lncRNA-based therapeutics is only in its infancy, however, their appealing properties such as their dysregulation in malignancies, tissue-specific expression, and tight-transcriptional control make them promising candidates for cancer therapeutic targeting. In parallel, targeting a specific lncRNA using antisense oligonucleotides (ASOs) which triggers RNases H-mediated degradation has made lncRNA-based therapeutics feasible [211]. These lncRNA-targeting ASOs can be chemically modified using 2′-O-Me or LNAs to improve their stability in vivo [211]. Taken together, growing knowledge of ncRNAs in EMT and metastasis and further improvements of nucleotide modifications and in vivo delivery systems may ultimately enable the translation of this novel knowledge into clinical practice.

## Concluding remarks and future perspectives

EMT is a highly dynamic cellular program that enables epithelial cells to transiently acquire a mesenchymal phenotype. Instead of oscillating between a complete epithelial and a complete mesenchymal state, cells can reside at an intermediate state, displaying both epithelial and mesenchymal features [43, 212]. Partial EMT in a cancerous context has been observed in experimental models and clinical settings [213, 214]. These studies demonstrated that the high plasticity of partial EMT enables tumor cells to adapt to a stressful environment during circulation, colonizing and formation of metastases, emphasizing the pivotal function of partial EMT in metastasis [213]. Although the detailed molecular mechanisms underlying partial EMT still remain elusive, mathematic modeling and experimental approaches demonstrated that double-negative feedback loops such as the miR-34/SNAIL and the miR-200/ZEB loops function as the ‘motor of cellular plasticity’ to control epithelial-hybrid-mesenchymal transitions [215–217]. Furthermore, cancer cells at the invasive front of solid tumors may undergo EMT, dependent on the link between miR-205 and ZEB1/2 [218]. These observations suggest that miRNAs are highly involved in regulating partial EMT and tumor metastasis, making them attractive candidates for diagnostic and therapeutic approaches. However, further investigation of the link between partial EMT and various forms of ncRNAs will be required to employ them in the development of novel therapeutic and diagnostic tools.

Moreover, tumor cells with an intermediate EMT phenotype, tend to develop CSC-like characteristics [43, 212, 219]. CSCs represent a minor subpopulation of tumor cells that can evade the immune system and are more resistant to most conventional therapeutics. Besides, CSCs may harbor tumor-propagating and metastatic capabilities [3]. Thus, given the crucial role of EMT and CSCs in tumor progression and the frequent development of resistance to various therapeutics, yielding novel therapeutic approaches designed to target the EMT process is promising. Anti-EMT therapeutic approaches can be achieved via inhibiting EMT initiation, targeting cancer cells that have undergone EMT as well as induction of an EMT reversal program in cancer cells [219]. Application of these strategies, using small-molecule inhibitors and biological agents, such as monoclonal antibodies, have been reviewed elsewhere and promisingly several of them has reached clinical trials or even received FDA approval [219]. ncRNA-based therapeutics can also be used to target EMT and may have distinct advantages over other compounds [13], for instance by targeting several RNAs simultaneously, which may lead to less acquired therapy resistance and better clinical outcomes. Additionally, as the ncRNA-based therapeutics are mainly based on base-pairing, the potential therapeutics can be easily designed and synthesized to be tested in preclinical and clinical models, while high-throughput screening and structure-based strategies should be employed for potential small molecule inhibitors and biological agents [13]. Despite these advantages, the major challenges in developing ncRNA-based therapeutics include successful delivery to the target cells while preventing nuclease-mediated degradation, avoiding immune system activation, and reducing off-target effects [13].

As summarized in this review, ncRNAs regulate EMT at multiple levels. Although the role of miRNAs in regulating EMT has been intensively investigated, the impact of other ncRNAs is still far from being fully understood and several controversies are associated with the lncRNA and circRNA research fields. In particular, concerning studies suggesting that lowly expressed lncRNAs or circRNAs containing only one or few miRNA binding sites function as efficient miRNA sponges [16, 34, 220]. More research is needed to gain a deeper understanding of the roles of ncRNAs as EMT-regulators in cancer, which may facilitate the development of novel diagnostic and therapeutic tools to impair metastasis and resistance to therapy.

## Data Availability

Data sharing is not applicable to this article as no datasets were generated or analyzed during the current study.

## Abbreviations

3′UTR: 3′ untranslated region
A-to-I: Adenosine-to-inosine
ASO: Antisense oligonucleotide
ccRCC: Clear cell renal cell carcinoma
ceRNA: Competitive endogenous RNA
circRNA: Circular RNA
CRC: Colorectal cancer
CSC: Cancer stem cell
EMT: Epithelial-mesenchymal transition
ESCC: Esophageal squamous cell carcinoma
HCC: Hepatocellular carcinoma
LNA: Locked nucleic acid
lncRNA: Long non-coding RNAs
MET: Mesenchymal-epithelial transition
miRNAs: microRNAs
ncRNA: Non-coding RNA
NSCLC: Non-small cell lung cancer
OSCC: Oral squamous cell carcinoma
piRNA: P-element Induced WImpy testis (PIWI)-interacting RNA
R-SBE: RNA-Smad binding element
snoRNA: Small nucleolar RNA
SNORA: H/ACA box snoRNA
SNORD: C/D box snoRNA
tRF: Transfer RNA-derived RNA fragment
tRNA: Transfer RNA
